# Altered Experienced Thermoregulation in Depression—No Evidence for an Effect of Early Life Stress

**DOI:** 10.3389/fpsyt.2021.620656

**Published:** 2021-07-21

**Authors:** Sarina von Salis, Ulrike Ehlert, Susanne Fischer

**Affiliations:** Clinical Psychology and Psychotherapy, Institute of Psychology, University of Zurich, Zurich, Switzerland

**Keywords:** autonomic nervous system, depression, fatigue, sweat, temperature, thermoregulation, stress, sleep

## Abstract

**Objectives:** Accumulating evidence suggests that individuals with depression are characterised by difficulties in thermoregulatory cooling. The aim of this study was to investigate, for the first time, whether depressed individuals are aware of these alterations, what their physical consequences are and whether they may be rooted in early life stress.

**Methods:** A total of *N* = 672 medically healthy individuals from the general population were recruited to participate in an online survey. Participants were divided into depressed vs. non-depressed using the Patient Health Questionnaire. Experienced autonomic and behavioural thermoregulation as well as vigilance problems in response to temperature increases were assessed by the Experienced Temperature Sensitivity and Regulation Survey. The Childhood Trauma Questionnaire was administered to assess early life stress.

**Results:** Controlling for age, sex, body mass index, and physical activity, depressed vs. non-depressed individuals did not differ in their experienced autonomic and behavioural responses to temperature increases. However, the depressed individuals reported comparably greater difficulties in concentrating and drowsiness/fatigue in warm environments (*p* = 0.029), during physical exertion (*p* = 0.029), and during stress (*p* < 0.001). There were no differences in the experienced thermoregulation between depressed individuals with vs. without early life stress.

**Conclusions:** Depressed individuals experienced more severe physical impairments (i.e., greater vigilance problems) in response to intense warmth when compared to non-depressed individuals. These differences were not attributable to comorbid illnesses, the intake of medication, or physical deconditioning. Further enquiries in clinical populations are warranted to investigate to what extent the observed alterations map onto specific symptoms of depression (e.g., sleep disturbances).

## Introduction

Accumulating evidence suggests that individuals with depressive disorders are affected by altered thermoregulation, and in particular by difficulties in thermoregulatory cooling [see (1) for a review]. Thermoregulation can be governed by the autonomic nervous system and/or by behaviour. *Autonomic* thermoregulation is governed by the preoptic area of the hypothalamus, and, in the case of temperature increases, involves the withdrawal of vasoconstriction, the initiation of vasodilation, and sweating (2). *Behavioural* thermoregulation is presumably orchestrated by the insula (3), and, in the case of temperature increases, includes changing locations, taking off layers of clothing, or turning on air conditioning.

In persons with depressive disorders, attenuated functioning of the sympathetic part of the autonomic nervous system has been found as compared with healthy controls [see (4)]. Furthermore, there is evidence for reduced sweating (5) as well as increased oral temperature [e.g., (6)]. These findings support the notion of impaired thermoregulatory cooling in this population. However, to date, nothing is known about whether depressed individuals are aware of these alterations, how they are affected by them, and what factors contribute to this phenomenon.

The aims of this study were to investigate whether depressed individuals experience alterations in autonomic and behavioural thermoregulation; and to explore the physical consequences and potential origins thereof. With regard to potential origins, the role of early life stress is of particular interest: Animal studies have provided direct evidence that early life stress exerts effects on the thermoregulatory system (7). This is in line with an extensive literature on stress-induced thermoregulatory impairments in both animals and humans [see e.g., (8) for a review]. Furthermore, studies in humans suggest that it is possible for these effects to be mediated by alterations in the autonomic nervous system [e.g., (9)]. Based on these and the above findings, we tested the following hypotheses: (a) Depressed vs. non-depressed individuals will report reduced autonomic thermoregulation, increased behavioural thermoregulation (by way of compensation), and greater vigilance problems in response to temperature increases; (b) these alterations will be more pronounced in individuals with vs. without early life stress.

## Methods

### Sample and Procedures

The data for the present study were collected as part of an online survey on early life experiences and thermoregulation, which comprised *N* = 1,301 German-speaking individuals from the general population. Participants were recruited by means of posts on websites, social media, and flyers. Exclusion criteria for the present study were: age <18, self-reported physical diseases or mental disorders (except depression), medication intake, high-risk alcohol consumption (≥2 standard units per day for women, ≥4 for men), recreational drug use, and regular smoking. The final sample included *N* = 672 individuals. The study protocol was approved by the ethics committee of the Faculty of Arts at the University of Zurich and electronic informed consent was obtained from all participants.

### Measures

*Self-reported depression* was assessed using the German version of the eight-item Patient Health Questionnaire [PHQ; (10)], which reflects eight of the nine symptoms (excluding suicidal ideation) of a major depressive episode as described in the DSM-5. Summing all items results in a score between 0 and 24. A score of 10 or higher is considered as suggesting a high probability of major depressive disorder and was thus used to divide participants into depressed vs. non-depressed.

*Experienced thermoregulation* was assessed using the Experienced Temperature Sensitivity and Regulation Survey [ETSRS; (11)]. The ETSRS assesses experienced thermosensation and thermoregulation and covers inputs to the thermal system (e.g., warm environments, physical exertion, and stress) and readouts of the thermal system (e.g., behavioural, autonomic, and vigilance responses), among several other aspects. Each of the original 102 items is rated on a 7-point bidirectional scale ranging from much less (−3) to much more (+3), with a neutral category of “like everyone else” (0). For the online survey, a 26-item German version of the ETSRS was created using a forward-backward translation procedure. This version maintains the original structure of the ETSRS, but is more condensed, such that, for instance, the items “Compared to others, a warm environment gives me warm hands/warm feet/a warm head/a warm trunk,” are collapsed into “Compared to others, a warm environment makes my body feel warm” (the German short version of the ETSRS is available from the authors upon request). For the purpose of the present study, only the seven items covering behavioural, autonomic, and vigilance responses to temperature increases were relevant.

*Early life stress* was measured using the German version of the Childhood Trauma Questionnaire [CTQ; (12)]. This 25-item scale distinguishes between five types of childhood trauma: emotional abuse, physical abuse, sexual abuse, emotional neglect, and physical neglect. The presence of moderate to severe early life stress was assumed if respondents scored at or above specific validated cut-offs on at least one of the five subscales (13).

### Statistical Analysis

Assuming medium-sized effects, G^*^Power analysis yielded a necessary sample size of *N* = 90 when setting the alpha error at 0.05 and power at 0.8. One-way independent ANCOVAs were performed to compare depressed and non-depressed individuals regarding thermoregulation (one-tailed significance testing). Covariates for all analyses were selected a priori and included sex, age, body mass index (BMI), and physical activity. ANOVAs were conducted in order to contrast depressed individuals with vs. without early life stress (two-tailed significance testing). All analyses were conducted using SPSS (version 25) and *p*-values were adjusted for multiple testing.

## Results

### Sample Characteristics

Sample characteristics are provided in Table 1. *N* = 101 individuals were depressed and *n* = 571 were non-depressed. Within the former group, *n* = 45 individuals had experienced early life stress and *n* = 56 had not. Depressed individuals were significantly more likely to be female and of younger age. In addition, they showed a lower educational attainment and had a lower annual household income than did non-depressed individuals. Moreover, they engaged in fewer hours of physical activity per week, but did not differ from non-depressed individuals regarding their BMI.

**Table 1:** Sociodemographic and lifestyle characteristics in depressed (*n* = 101) vs. non-depressed (*n* = 571) individuals.

	**Non-depressed**	**Depressed**	**Statistics**
Sex			Fisher's exact-test, *p* = 0.014
Male [*n* (%)]	152 (27%)	14 (14%)	
Female [*n* (%)]	416 (73%)	87 (86%)	
Other [*n* (%)]	3 (0%)	0 (0%)	
Age [*Mdn* (*IQR*)]	25 (15)	22 (5)	*t* = 7.147, *p* < 0.001
Body mass index [*Mdn* (*IQR*)]	22.2 (4)	22.6 (5)	*t* = −0.690, *p* = 0.491
Education [*n* (%)]			Fisher's exact test, *p* = 0.001
No education	1 (0%)	4 (4%)	
Mandatory school	6 (1%)	1 (1%)	
Apprenticeship	32 (6%)	3 (3%)	
High school	286 (50%)	69 (68%)	
University degree	246 (43%)	24 (24%)	
Household income [*Mdn* (Min–Max)]	4 (1–7)	3 (1–7)	*U* = 24,388.000, *p* = 0.015
Physical activity [*Mdn* (Min–Max)]	2 (1–4)	2 (1–4)	*U* = 25,324.500, *p* = 0.038

*Mdn, median; IQR, interquartile range; Min, minimum; Max, maximum; annual household income coded as 1 = no income, 2 = <30,000 CHF, 3 = 30,001–50,000 CHF, 4 = 50,001–100,000 CHF, 5 = 100,001–150,000 CHF, 6 = 150,001–200,000 CHF, and 7 = >200,000 CHF; physical activity per week coded as 1 = <1 h, 2 = 1–3 h, 3 = 4–6 h, and 4 = >7 h*.

**Figure 1:**
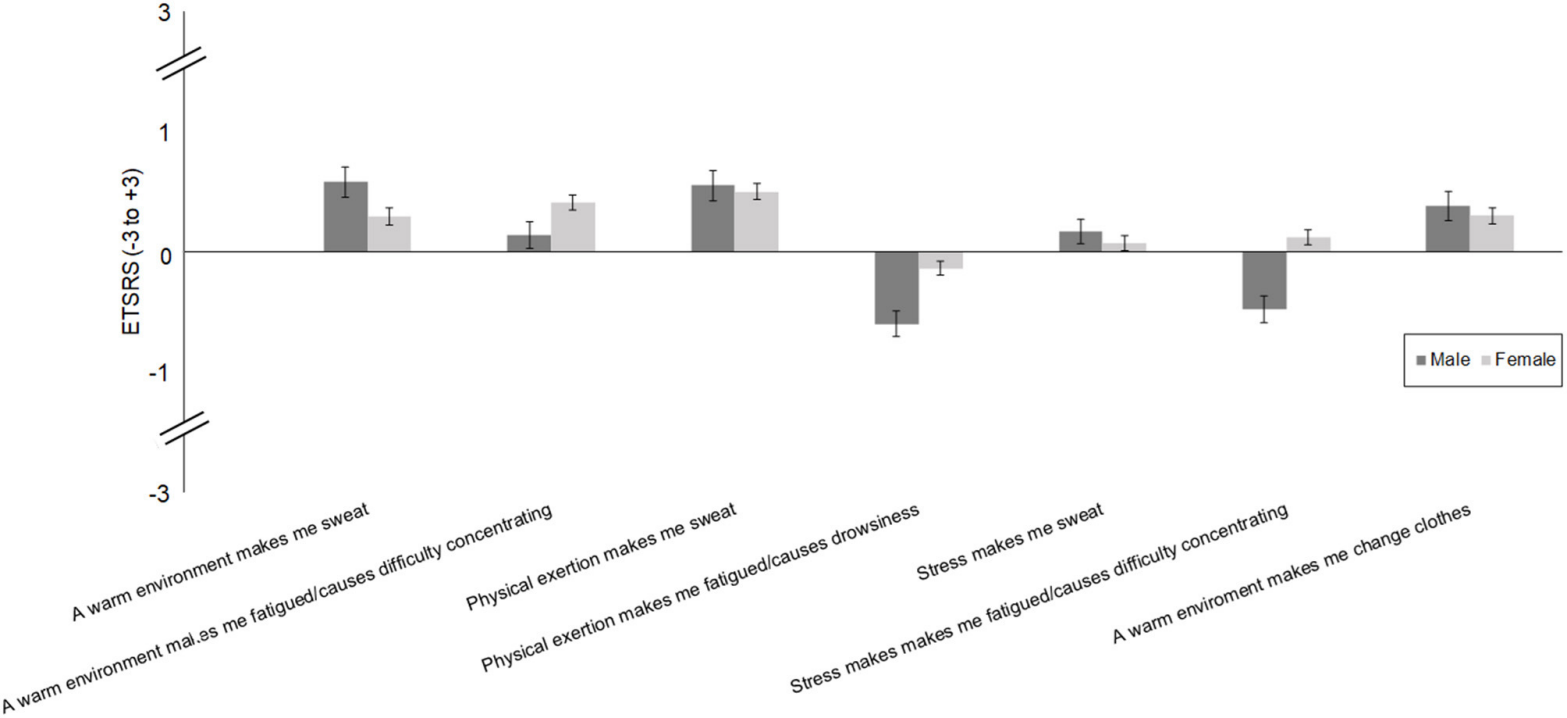
Autonomic and behavioural thermoregulation [Experienced Temperature Sensation and Regulation Survey (ETSRS)] in individuals with self-reported male vs. female sex.

For descriptive purposes, the items of the ETSRS according to self-reported male or female sex are provided in Figure 1.

### Depressed vs. Non-depressed Individuals

**Figure 2:**
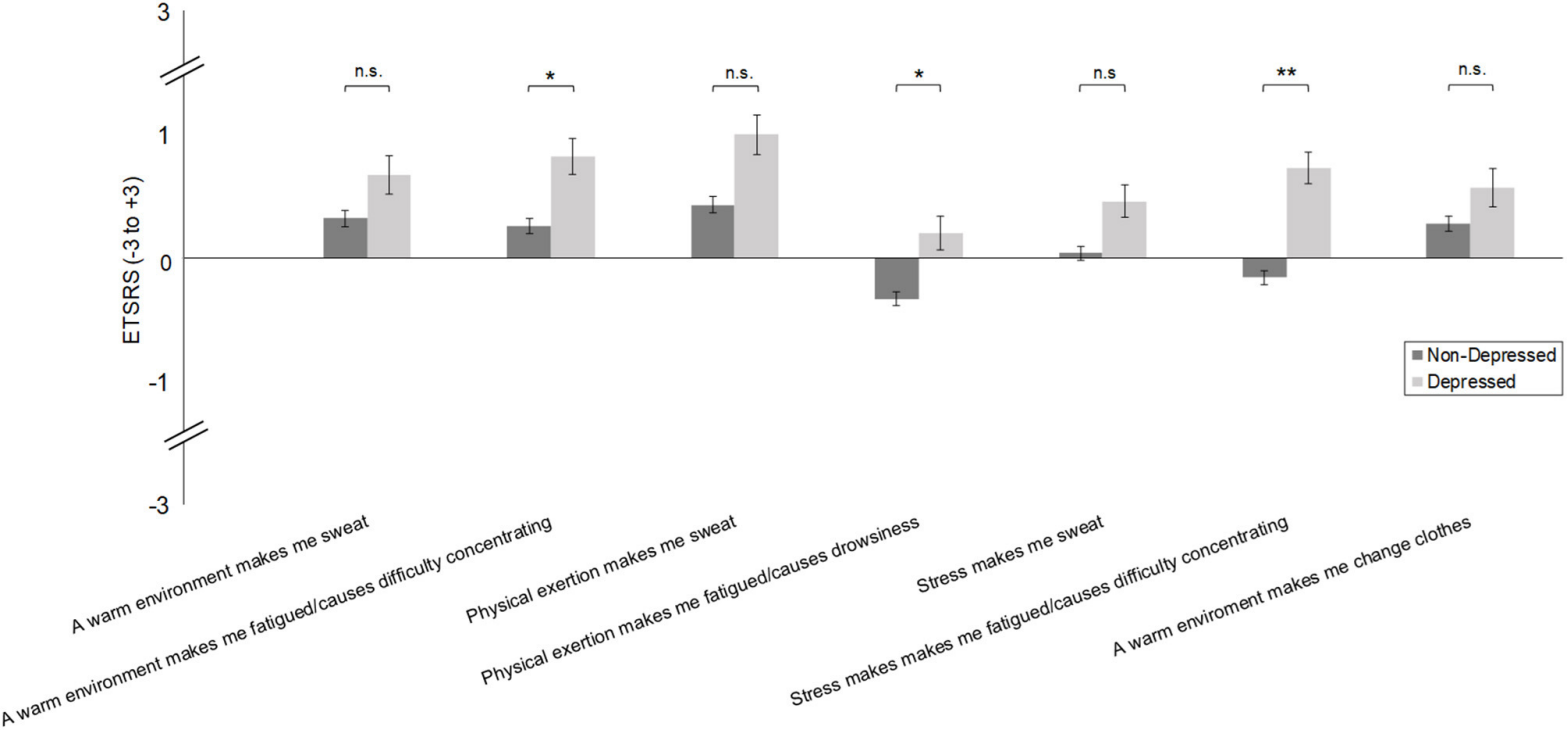
Autonomic and behavioural thermoregulation [Experienced Temperature Sensation and Regulation Survey (ETSRS)] in depressed vs. non-depressed individuals [Patient Health Questionnaire (PHQ)]. Bars represent mean values and error bars display the standard error of the means. *p < 0.05. **p < 0.01. n.s. = not significant.

Controlling for age, sex, BMI, and physical activity, depressed vs. non-depressed individuals did not differ in their experienced autonomic responses to temperature increases [warm environments *F*_(1,666)_ = 1.151, *p* = 0.284; physical exertion *F*_(1,666)_ = 4.318, *p* = 0.057; stress *F*_(1,666)_ = 4.460, *p* = 0.057; see Figure 2]. However, they reported greater vigilance problems in warm environments [*F*_(1,666)_ = 5.519, *p* = 0.029, partial η^2^ = 0.007], during physical exertion [*F*_(1,666)_ = 4.766, *p* = 0.029, partial η^2^ = 0.007], and during stress [*F*_(1,666)_ = 28.150, *p* < 0.001, partial η^2^ = 0.041]. Depressed vs. non-depressed individuals did not differ in their behavioural responses to warm environments [*F*_(1,666)_ = 2.576, *p* = 0.109].

### Role of Early Life Stress

There were no differences in the experienced thermoregulation between depressed individuals with vs. without early life stress (vigilance problems in warm environments *F*_(1, 89.858)_ = 3.702, *p* = 0.174; vigilance problems during physical exertion *F*_(1, 84.568)_ = 0.200, *p* = 0.886; vigilance problems during stress *F*_(1, 77.892)_ = 0.021, *p* = 0.886).

## Discussion

This study yielded two main findings. First, when compared to non-depressed individuals, depressed individuals did not differ from healthy controls in their experienced autonomic and behavioural thermoregulation during temperature increases, whilst at the same time reporting more severe physical consequences. Second, the degree of the physical consequences did not differ between depressed individuals with vs. without early life stress.

The finding that depressed individuals did not differ from healthy controls in their experienced autonomic responses to warmth appears to contradict physiological studies, which have observed comparably attenuated sympathetic functioning (4) and sweating in depression (5) as well as increased oral temperature (6). This dissociation between physiological and psychological measures of thermoregulation mirrors the observation that individuals with major depressive disorder tend to exhibit comparably elevated heat pain thresholds (i.e., reduced pain perception) in combination with reports of equal or even greater pain intensity (14). One potential explanation for this is impaired interoceptive awareness. Indeed, depressed states have repeatedly been shown to be paralleled by a reduced capability to accurately perceive internal states or processes, such as heart beats [e.g., (15)]. However, further research into the correspondence of physiological and subjective thermoregulatory measures in depression is warranted. Importantly, in the present study, depressed individuals indicated more physical consequences of warmth (i.e., greater difficulties in concentrating and greater fatigue). Together, these findings suggest that these individuals' efforts at thermoregulatory cooling are insufficient to protect them from physical discomfort when their body temperature increases.

Interestingly, there was also no indication of (compensatory) increases in behavioural responses to warm environments. One explanation for this may lie in the concept of learned helplessness (16), insofar as depressed individuals may passively endure the unpleasant experience of intense warmth rather than actively attempt to counteract it. Alternatively, it is conceivable that group differences would have become apparent when referring to conditions of more extreme temperature increases (e.g., during physical activity or stress), which were not assessed in the present study. Further research is thus warranted to investigate behavioural thermoregulation in depression in additional contexts. This is particularly important given that altered autonomic thermoregulation may map directly onto sleep disturbances, and behavioural measures may prove useful in counteracting these (17).

The present study was unable to identify a difference in thermoregulatory functioning between depressed individuals with and without early life stress. This finding is in contrast to a study investigating this relationship in rats, which demonstrated that maternal separation was linked to a reduced recruitment of autonomic thermoeffectors and a hyperthermic state during warmth exposure (7). Notably, in the latter study, all animals exposed to maternal separation also developed depressive-like behaviour. It is thus possible that the depressive symptoms, rather than the early life stress, were the cause of the thermoregulatory alterations. Against this, emerging evidence in humans points to alterations in thermoregulatory mediators, such as the sympathetic nervous system [e.g., (9)] or the hypothalamic-pituitary-thyroid axis [e.g., (18)], in the aftermath of early life stress. Similarly, early life stress has been found to affect serotonergic signalling [e.g., (19)], another system implicated in thermoregulation. Hence, further research is needed to disentangle the role of stress and depression in thermoregulatory alterations.

This is the first study to investigate experienced thermoregulation in depressed individuals and the first to explore the role of early life stress in this context. A further strength of the study lies in the large sample size, which allowed us to eliminate the influence of a number of cofounding factors when contrasting depressed vs. non-depressed individuals (e.g., medication intake). A limitation is the recruitment *via* websites, social media, and flyers, which may have limited the representativeness of our sample. A further limitation is that depression was assessed by means of a self-report measure. Despite the high sensitivity of the PHQ in detecting major depressive episodes (20), it is thus unclear whether the individuals in this study suffered from clinical levels of depression. Finally, due to the cross-sectional design of the study, it is not possible to establish the temporal order between thermoregulatory alterations and depressive symptoms.

Although the effects reported in the present study are small, it is important to consider potential clinical implications of the findings. Given that body temperature is a direct contributor to sleep propensity (21) and that alterations in thermoregulatory functioning are associated with sleep disorders (17), ineffective thermoregulation might directly contribute to insomnia, as is frequently present in major depressive disorder. In line with this notion, Harding et al. (22) showed that skin warming recruits a hypothalamic circuit that functions to promote body cooling and sleep in mice. Further research in depression is warranted to investigate whether a dysfunction of this circuit may be a mediator of the relationship between altered thermoregulation and sleep.

Moreover, the current findings suggest that some of the most common symptoms of major depressive disorder, namely cognitive problems and fatigue, may be exacerbated by intense warmth. Interestingly, a previous study demonstrated that one single session of a warmth-based treatment was capable of reducing body temperature in patients with major depressive disorder, while at the same time leading to significant mood improvements (23). The mechanisms underlying this effect remain largely unclear, but may include changes in the immune system, which has repeatedly been found to be altered in depression (24).

In sum, the results of the present study indicate that depressed individuals experience greater physical impairment in response to warmth. Importantly, these differences are not the consequence of any physical illness, the intake of medication, or physical deconditioning. Further enquiries in clinical populations are now called for to investigate the extent to which the observed alterations are linked to sleep disturbances and other symptoms of depression.

## Data Availability Statement

The raw data supporting the conclusions of this article will be made available by the authors, without undue reservation.

## Ethics Statement

The study was approved by the ethics committee of the Faculty of Arts at the University of Zurich. The participants provided their written (online) informed consent to participate in this study.

## Author Contributions

SF conceived the study. SF and SS designed the study and drafted the article. SS analysed the data. SS, SF, and UE interpreted the data. UE revised the article critically for important intellectual content. All authors have approved the final article.

## Conflict of Interest

The authors declare that the research was conducted in the absence of any commercial or financial relationships that could be construed as a potential conflict of interest.
